# Standard Triple Therapy versus Sequential Therapy in *Helicobacter pylori* Eradication: A Double-Blind, Randomized, and Controlled Trial

**DOI:** 10.1155/2015/818043

**Published:** 2015-05-04

**Authors:** Jaime Natan Eisig, Tomás Navarro-Rodriguez, Ana Cristina Sá Teixeira, Fernando Marcuz Silva, Rejane Mattar, Decio Chinzon, Christiane Haro, Márcio Augusto Diniz, Joaquim Prado Moraes-Filho, Ronnie Fass, Ricardo Correa Barbuti

**Affiliations:** ^1^Division of Gastroenterology and Clinical Hepatology, Hospital das Clínicas da FMUSP, 05493-900 São Paulo, SP, Brazil; ^2^Division of Gastroenterology and Hepatology, MetroHealth Medical Center, Case Western Reserve University, Cleveland, OH 44109, USA

## Abstract

*Aim*. To compare 10-day standard triple therapy versus sequential therapy as first-line treatment in patients infected with *H. pylori*. *Methods*. One hundred *H. pylori* positive patients (diagnosed by rapid urease test and histology), with average age of 47.2, M/F = 28/72, were randomized to receive either standard triple treatment (TT) as follows: lansoprazole 30 mg, clarithromycin 500 mg, and amoxicillin 1 g, b.i.d. for ten days, or sequential treatment (ST) as follows: lansoprazole 30 mg, amoxicillin and placebo 1.0 g b.i.d for the first five days, followed by lansoprazole 30 mg, clarithromycin 500 mg, and tinidazole 500 mg b.i.d, for the remaining five days. Eradication rates were determined 60 days after treatment by urease, histology, or ^13^C-urea breath test. *Results*. In intention to treat (ITT) analysis, the rate of *H. pylori* eradication in the TT and ST groups was the same for both regimens as follows: 86% (43/50), 95% CI 93,3 to 73.4%. In Per protocol (PP) analysis, the rate of *H. pylori* eradication in the TT and ST groups was 87.8% (43/49), 95% CI 94,5 to 75.3% and 89.6% (43/48), 95% CI 95,8 to 77.3%, respectively. *Conclusions*. In Brazil, standard triple therapy is as equally effective as sequential therapy in eradicating *Helicobacter pylori* patients. This study was registered under Clinical Trials with number ISRCTN62400496.

## 1. Introduction

There are several therapeutic regimens to eradicate* H. pylori*, involving bismuth, clarithromycin, amoxicillin, furazolidone, nitroimidazole compounds, quinolones, and proton pump inhibitors, in different combinations [1–6].

The triple therapy (proton pump inhibitor, clarithromycin, and amoxicillin) for seven days has been the most widely used regimen, recommended by many national and international consensus meetings [7, 8]. However, over the past several years a considerable drop in the eradication rates of* H. pylori* has been observed, reaching unacceptable levels (less than 80%) [9, 10]. This phenomenon has been reported by authors from all over the world due to a considerable increase in the prevalence of resistance to clarithromycin and metronidazole [10, 11]. In Brazil, this is also the situation, though in smaller scale [12, 13], because the susceptibility of strains of* H. pylori* to clarithromycin is still high [14–16]. The resistance to* H. pylori* varies from one country to another and also in different regions of the same country [17].

In Europe and Asia, a new therapeutic regimen has been used for a few years. It is called sequential therapy, which consists of a double scheme, with a proton pump inhibitor + amoxicillin for five days, followed by a triple therapy with proton pump inhibitor, clarithromycin, and tinidazole for five additional days. The sequential therapy achieves around 90–94% [18–21] eradication rates. These results, which although are already decreasing in effectiveness [22], have not yet been documented in Latin America [23].

In Brazil, we have not heard of studies using this therapy as the first choice. The aim of this study was to compare the eradication rates of* H. pylori* using sequential therapy versus triple therapy over a period of ten days.

## 2. Methods

### 2.1. Study Design

This is a randomized, double-blind, prospective trial, performed from October 2012 to December 2013, which included patients from the Gastroenterology Department at the University of São Paulo, School of Medicine, Clinical Hospital. Patients at least 16 years old, who underwent an upper endoscopy due to dyspeptic symptoms and were found to have* H. pylori* infection confirmed by the rapid urease test and histology, were enrolled into this study. None of the patients received previous eradication treatment. Exclusion criteria included previous treatment for* H. pylori* and previous use of proton pump inhibitors, antibiotics, or chemotherapy in the four weeks that preceded the beginning of the trial. Patients who had undergone gastrectomy or had history of complicated ulcers (Forrest I and Forrest II), pregnant or breastfeeding women, and patients with consumptive diseases and with uncompensated kidney or heart failure were excluded as well. The study was performed in accordance with the Declaration of Helsinki and was approved by the institutional Ethics Review Board for clinical research. All patients signed an informed consent form. Patients whose* H. pylori* was not eradicated underwent retreatment with another therapeutic regimen.

Patients were randomized into two groups.

- Triple therapy (TT) for 10 days (30 mg lansoprazole, 500 mg clarithromycin, and 1.0 g amoxicillin, each administered twice a day).
- Sequential therapy (ST) for 10 days (30 mg lansoprazole and 1.0 g amoxicillin and placebo, each administered twice a day for five days, followed by 30 mg lansoprazole, 500 mg clarithromycin, and 500 mg tinidazole, each administered twice a day for the remaining five days).

An independent researcher who was in charge of concealing the medication was responsible for generating a computer-based sequence of random numbers. For each group of patients were prepared pill boxes containing the medications and placebo indistinguishable from active medicine.

### 2.2. Procedures

Patients with dyspeptic symptoms underwent an upper endoscopy.* H. pylori* infection was determined by the rapid urease test [24] and histology [25], using gastric mucosal biopsies of the antrum and body. Patients with positive results in the two methods were included in the trial.* H. pylori* eradication was assessed at least two months after the end of the treatment by urease, histology, and ^13^C-urea breath test in patients with peptic ulcer [26]. In functional dyspepsia patients, eradication was confirmed only through the ^13^C-urea breath test. All patients were advised to suspend treatment with proton pump inhibitors or H_2_ receptor antagonists, at least ten days prior to* H. pylori* testing.

The secondary goal of this study was to assess patients' adherence to treatment and possible adverse effects. Patients' adherence was determined by using capsule counting and considered satisfactory when more than 90% of the pills were taken. No questionnaires were used in this study.

This study was registered under Clinical Trials with number ISRCTN62400496.

### 2.3. Statistical Analysis

Sample size was calculated using the Fisher exact test with expected eradication rates of 75% and 95% for TT and ST, respectively, considering an 80% power level and 5% significance level.

Descriptive analysis was presented as mean (±standard deviation) for quantitative variables and absolute numbers (percentages) for qualitative variables. The Mann-Whitney test for quantitative variables and Fisher's exact test for qualitative variables were used to compare the two groups as well as the 95% confidence intervals for the difference between triple therapy and sequential therapy eradication rates.

Intention to treat (ITT) and per protocol (PP) analysis were considered. A *p* value <0.05 indicates statistical significance.

## 3. Results

### 3.1. Patients

One hundred patients with proved* H. pylori* infection were included in the trial. The baseline demographic and clinical characteristics of patients were similar in the 2 groups (Table 1).

Three patients who were excluded from the PP analysis are as follows: two discontinued because of adverse events (epigastric pain) and one was lost to follow-up.

All the patients in both groups adhered to the treatment (>90% of the medication was taken).

### 3.2. Clinical Efficacy

The eradication rates are shown in Table 2. There was no significant difference in the eradication rates between the Standard Triple Therapy and Sequential Therapy groups, in ITT and PP analysis.

Eighty-five patients were diagnosed with chronic gastritis and 15 with peptic ulcer disease (gastric ulcer or duodenal ulcer). Fifteen, out of the 97 patients who completed the study, had peptic ulcers and 82 had chronic gastritis; of these, 6 patients were using omeprazole, because of dyspeptic symptoms. The overall eradication rates in patients with chronic gastritis and those with peptic ulcer disease were 89.4% and 86.7% in the ITT analysis and 89.0% and 86.7% in PP analysis, respectively. There was no (ITT: *p* = 0.699; PP: *p* = 0.677) significant difference in the eradication rate between patients with chronic gastritis and those with peptic ulcer disease.

### 3.3. Adverse Events

Both treatments were well tolerated. There was no serious adverse event. Only two patients discontinued treatment because of adverse events (epigastric pain), one in the TT group and another in the ST group.  Table 3 shows the side effects.

## 4. Discussion

The results of this trial demonstrated that* H. pylori* eradication rates were similar for both groups. No significant difference in eradication rates was found between patients with chronic gastritis and those with peptic ulcers. Also, we have not seen important adverse effects, and only two patients had to stop treatment due to epigastric pain (one in each group). Other symptoms such as nausea, diarrhea, vomiting, heartburn, bloating, headache, and bitter taste were of mild intensity and similar in frequency in both groups. Although not statistically significant, a greater percentage of adverse events were observed in the sequential therapy (48% × 34,6%, *p* = 0.219).

Several studies in the literature have shown that the effectiveness of the triple therapy has been declining over the past years [27], mainly due to an increase in clarithromycin resistance, especially in the USA and Europe [28, 29]. For this reason, several other regimens have been assessed. A commonly used scheme is sequential therapy, which consists of the administration of PPI and amoxicillin during the first stage and PPI + clarithromycin and imidazolium in the second stage, with eradication rates greater than 90% [18, 20]. In view of the latter results, sequential therapy has been recommended as the preferred treatment to eradicate* H. pylori,* especially in Europe.

In Brazil, we have not tested sequential therapy as an alternative regimen to triple therapy. Because of the high prevalence of* H. pylori* infection in Brazil, it is important to have other therapeutic regimens, which should be more effective, accessible to the population, with good adherence, fewer adverse effects, and low cost.

Unlike studies from other countries, our trial has not shown significant difference in the eradication rates between the two regimens. As previously mentioned, resistance to antibiotics, especially clarithromycin, is the main reason for the decrease in* H. pylori* eradication rates. However, it is important to know that this resistance varies from country to country and from region to region in the same country [17, 30]. This variation in resistance can influence the efficacy of* H. pylori* therapeutic regimens. In Brazil, resistance to clarithromycin is still relatively low, while to metronidazole, it is very common [12–14].

Similar results were also found in other studies that were performed in Latin America [23, 31].

Another relevant factor to be considered is the cost of treatment. In Brazil, triple therapy is available in kits, with three drugs in the same packaging, which makes it easier for the patients to adhere to treatment. The cost of the triple therapy kit is U$ 60,00 on average, while the cost of sequential therapy is approximately U$ 130,00, more than twice as much. Moreover, there is no kit for sequential therapy.

Our trial has few limitations. We have not assessed* H. pylori* resistance to antibiotics through sensitivity tests, since it is a difficult test to perform in our community and, when available, it is still very costly for the patient.

New trials are necessary to assess if* H. pylori* in Brazil has a tendency to increase its resistance to clarithromycin. Until now, we have not observed these epidemiological trends, since our eradication rates in this trial were the same as we observed in a previous study that showed 88,8% and 82,7% eradication rates in peptic ulcer and functional dyspeptic patients, respectively [16].

## 5. Conclusion

In conclusion, our findings suggest that sequential therapy has not proven to be superior over triple therapy in eradicating* H. pylori* infection. Since it has satisfactory eradication rates, low cost, and less complexity for the patient, triple therapy is still a good first-line regimen to eradicate the* H. pylori* infection in Brazil.

## Figures and Tables

**Table 1:** Demographic and clinical characteristic of the patients.

	Triple therapy (*n* = 50) *N*/%	Sequential therapy (*n* = 50) *N*/%	*p* value
Mean age	50.2 ± 1.7	44.2 ± 1.6	0.011
Gender: female	32 (64%)	40 (80%)	0.118
Cigarette smoking	17 (34%)	11 (22%)	0.695
Alcohol drinking	6 (12%)	2 (4%)	0.269
Chronic gastritis	41 (82%)	44 (88%)	0.265
Duodenal ulcer	5 (10%)	4 (8%)	
Gastric ulcer	4 (8%)	2 (4%)	

**Table 2:** *Helicobacter pylori* eradication rates with triple and sequential therapy.

	Triple	95% CI	Sequential	95% CI	*p* value
Intention to treat	86.0% (43/50)	93.3–73.4	86.0% (43/50)	93.3–73.4	1
Per protocol	87.8% (43/49)	94.5–75.3	89.6% (43/48)	95.8–77.3	1

**Table 3:** Patients with self-reported adverse events during therapy.

Adverse event	Triple therapy	Sequential therapy	*p* value
	*N*/%	*N*/%	
Epigastric pain	3 (6.12%)	4 (8.33%)	0.715
Nausea	3 (6.12%)	5 (10.42%)	0.487
Diarrhea	5 (10.2%)	6 (12.5%)	0.759
Vomiting	2 (4.08%)	1 (2.08%)	1
Bloating	2 (4.08%)	3 (6.25%)	0.678
Heartburn	1 (2.04%)	0 (0%)	1
Headache	0 (0%)	1 (2.08%)	0.495
Bitter taste	1 (2.04%)	3 (6.25%)	0.362
